# The Cœlomic Microbiota Among Three Echinoderms: The Black Sea Cucumber *Holothuria forskali*, the Sea Star *Marthasterias glacialis,* and the Sea Urchin *Sphaerechinus granularis*

**DOI:** 10.3390/biology14040430

**Published:** 2025-04-16

**Authors:** Hélène Laguerre, Cyril Noël, Camille Jégou, Yannick Fleury, Patrick Le Chevalier

**Affiliations:** 1 Laboratoire de Biotechnologie et Chimie Marines, LBCM, EMR-CNRS 6076, University of Brest, F-29000 Quimper, France; helene@huitres-cadoret.fr (H.L.); camille.jegou@univ-brest.fr (C.J.);; 2 IFREMER-IRSI-Service de Bioinformatique (SeBiMER), 1625 Route de Sainte-Anne, F-29280 Plouzané, France; cyril.noel@ifremer.fr

**Keywords:** Echinodermata, *Holothuria forskali*, *Sphaerechinus granularis*, *Marthasterias glacialis*, microbiota, cœlomic fluid, V4 Metabarcoding, antibacterial activity, 16S rRNA

## Abstract

In this study, the bacterial communities of the cœlomic microbiota were characterized in three Echinoderms: the deposit feeder sea Cucumber *Holothuria forskali*, the herbivorous sea Urchin *Sphaerechinus granularis*, and the carnivorous sea Star *Marthasterias glacialis*. Samples were collected from the same habitat in the Glénan Archipelago (Brittany, France) at different times for 2 years. The cœlomic microbiota were analyzed by targeted metagenomic with V4-16S metabarcoding and by a culturable approach with the isolation of strains and antimicrobial activity assays. Most of the OTUs of the cœlomic microbiota were affiliated with the phylum Proteobacteria and, notably, five orders: Burkholderiales, Flavobacteriales, Alteromonadales, Vibrionales and Pseudomonadales. Significant differences were observed regarding richness, biodiversity and composition between species and sampling dates. They could be explained by sub-abundant taxa that represented the global diversity. Cœlomic microbiota also revealed shared and unshared bacterial communities, validating a potential “specific” microbiota among the three Echinoderm species. Moreover, significant variations of the microbiota occurred among the sampling dates, suggesting a plasticity and, thus, a potential selection of these microbiota. Finally, out of the 831 bacterial strains isolated from culturable microbiota, 20 strains exhibited antibacterial activities, most of them assigned to the genera *Shewanella, Pseudoalteromonas* and *Vibrio*.

## Simple Summary

Echinoderms play an important role in all marine ecosystems, as bioturbation and organic matter recycling animals. Moreover, exploration of their microbiota reveals interesting compounds for biotechnological issues such as antitumor, cytotoxic or antibacterial agents. The coelomic fluid constitutes a key compartment for immunity and homeostasis in Echinoderms, with specialized cells called coelomocytes, and also with the microbiota. The coelomic microbiota remains unexplored. In this study, we compared the coelomic microbiota of three Echinoderms: a sea urchin, a sea star and a sea cucumber, living in the same habitat in a marine-protected area (Brittany, France) by both 16S metabarcoding and culture-based approaches. The results highlighted the existence of a core coelomic microbiota to an echinoderm host, and varying according to time, 20 antibacterial strains were isolated from these microbiotas and constituted potential probiotic candidates.

## 1. Introduction

The Echinoderms are interesting organisms that taxonomically belong to the Deuterostomians within the Chordates and diverge from other marine invertebrates of the Bilateria Clade such as Molluscs, annelids or arthropods that belong to the Protostomians [1]. The taxon “Echinoderms” was first described in the 18th century and then classified into an independent phylum by Mortensen in the 20th [2] and divided in five classes: the Asteridea (sea stars), the Echinideae (sea urchins), the Crinoidea (crinoids), the Ophiuridea (brittle stars) and the Holothuridea (sea cucumbers).

These marine organisms, mostly benthic animals, are identified by morphological characteristics of the phylum, including a pentaradial symmetry and a cœlomic cavity filled with cœlomic fluid. This cœlomic fluid plays an important role in metabolism, immunity and homeostasis [3,4]. This immunity is provided by the coelomocytes that constitute cellular mediators for the immune response [5,6,7]. Moreover, this biological compartment contains a part of their microbiota that could be especially implicated in the immunity response with bacteria-producing antibacterial compounds [8,9,10,11,12,13]. Some of these antibacterial strains, isolated from marine invertebrates, could be used in aquaculture as probiotics to boost zootechnical performances [14,15,16,17,18] or to prevent diseases [19,20,21].

However, these culturable bacteria represent only a limited part of the total microbiota [22]. With the development of NGS technologies and the metabarcoding methods, research on microbiota has exploded [23,24,25,26], first on Sponges [27,28] and Corals [29,30], and then on Ascidians [31], Cnidarians [10,32] and Molluscs [33,34].

In Echinoderms, most of the studies explored the gut microbiota in commercial species, such as the Japanese sea cucumber *Apostichopus japonicus* [13,35,36] and the edible sea urchin *Paracentrotus lividus* [37]. To date, the cœlomic microbiota remains underexplored.

The analysis of the cœlomic microbiota of a sea star demonstrated a specific and selected microbiota compared to bacterial communities of ambient seawater [38], which was mostly composed of Proteobacteria, the order Alteromonadales (genus *Pseudoalteromonas*) [39] and the families *Flavobacteriaceae* and *Rhodobacteraceae* [38,40]. Moreover, the composition of these cœlomic microbiota exhibited specific and non-culturable families of bacteria. Otherwise, the existence of spatiotemporal variations and important inter- and intraspecific variations of this microbiota was also reported in Asteridea [40].

In Holothuridea, only a few studies have been conducted to define the culturable and non-culturable cœlomic microbiota [10,11,22,41,42]. In *A. japonicus*, the composition of cœlomic microbiota was dominated by *Proteobacteria* and, more precisely, by the taxa *Pseudoalteromonas*, *Shewanella* and *Flavobacteriaceae* [22,42], and the presence of unique bacteria from Epsilonproteobacteria and Rickettsiales has been reported [21]. In *Holothuria tubulosa* and *H. forskali*, from the NE Atlantic and in the Mediterranean Sea, the composition of the pooled microbiota of intestines and cœlomic fluid differed between species and from seawater and exhibited dominant OTUs from the *Flavobacteriaceae* [10]. In addition to the growing interest in describing the composition of bacterial communities using genomic tools, understanding the relationship between this microbiota and its host is essential.

Furthermore, the presence of bacteria in fluid compartments such as hemolymph and coelomic fluid in invertebrates is now well accepted by the scientific community. However, the role of this microbiota is still poorly understood: a microbial shield? Some bacteria already isolated from these biological fluids were capable of producing antimicrobial compounds. We therefore focused on the part of the coelomicrobiota exhibiting antibacterial activities. Thus, previous studies on culturable cœlomic microbiota showed a concentration around 10^5^ CFU/mL for *H. forskali* and permitted the isolation of antimicrobial and antifungal strains from both species [10,11].

This study aimed to explore the composition and diversity of the cœlomic fluid microbiota from three Echinodermata living in a same habitat in the NE Atlantic: the sea cucumber *Holothuria forskali* (Delle Chiaje 1823), the sea urchin *Sphaerechinus granularis* (Lamarck 1816) and the sea star *Marthasterias glacialis* (Linnaeus 1758). The cœlomic microbiota were analyzed by two complementary methods: 16S metabarcoding using Illumina MiSeq sequencing technology and the culturable approach with strains isolation and antibacterial screening.

## 2. Material and Methods

### 2.1. Sample Collection

**Figure 1:**
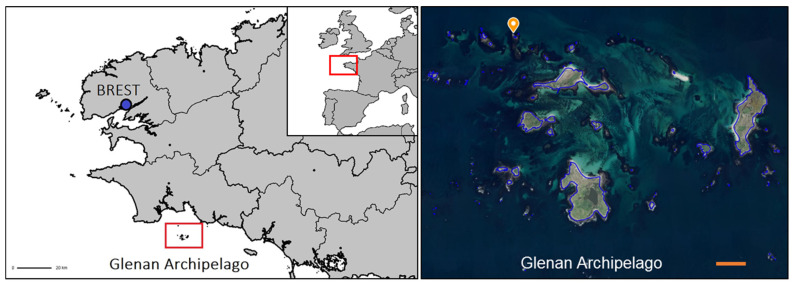
Sampling in the Glénan Archipelago (South Brittany, France). Sampling area and diving zone located with an orange point; scale bar in orange: 500 m (source: geoportail.gouv.fr).

Four collections were carried out over 2 successive years: 2 on 27 February 2019 and on 22 January 2020 (seawater temperature 10 °C) and on 5 June 2019 and on 10 June 2020 (seawater temperature: 15 °C). Animals were collected by scuba diving between 5 and 12 m in depth in the Glénan Archipelago (South Brittany, France, WGS84: 47°43′57.76″ N and 04°00′50.99″ W) (Figure 1). For each sampling period, 15 specimens were collected for each of the 3 species: *H. forskali, S. granularis* and *M. glacialis*. These 3 benthic species could be found mostly on vertical walls in the same habitat composed of rocky beds colonized with kelp (*Laminaria* sp. and *Sargassum* sp. principally) and encrusting algae. Animals were stored in distinct 50 L tanks filled with local seawater and directly transferred to the laboratory. Three samples of ambient seawater were collected in sterile 15 mL centrifuge tubes.

Animals were weighed and measured in the laboratory (Supplementary Figure S1). For each animal, two cœlomic fluid samples were obtained for the culturable and non-culturable analyses. Before each sample collection, the needle insertion area on the animal’s body surface was washed and disinfected using 70% ethanol. For *H. forskali*, cœlomic fluid was collected into the cœlomic cavity by introducing a 25G needle fixed on a 2.5 mL syringe through the body wall on the ventral side. For *S. granularis*, the needle was inserted in the body cavity by the oral pole of the sea urchin through the peristomial membrane. For *M. glacialis*, cœlomic fluid was removed by dissection of a branch of the sea star, and the cœlomic fluid was collected in sterile 15 mL tubes. For the non-culturable analysis, tubes were centrifuged at 13,000× *g* for 10 min to precipitate bacteria. Supernatants were removed, and the pellets were then stored at −20 °C before further analysis. For studies on culturable microbiota, samples were directly processed.

### 2.2. DNA Extraction, Amplification and Illumina MiSeq Sequencing

Bacterial DNA from pellets stored at −20 °C was extracted with the Qiagen DNA Stool mini Kit (Qiagen, Hilden, Germany) according to the manufacturer’s instructions (protocol for pathogen detection, with an initial lysis step at 95 °C). The products of DNA extraction were then amplified on the AnaEE Platform (Grenoble, France). Four replicates of each sample and four negative controls were performed for the randomized PCR, focused on the hypervariable region V4 of the 16S rDNA gene, using barcoded primers for bacteria: Bact02F (5′-GCCAGCMGCCGCGGTAA-3′) and rBact02R (5′-GGACTACCMGGGTATCTAA-3′) according to the Metafast protocol [43]. Sequencing was then performed with the technology Illumina MiSeq (2 × 250 pb) by Fasteris (Geneva, Switzerland). Finally, sequencing data were sent in the format fastq.gz for bioinformatics analysis.

### 2.3. Bioinformatics and Data Processing

After quality control and trimming on sequences, sequences were assigned to samples with ngsfilter from Obitools [44]. Then, sequencing data were analyzed using Frogs 3.1.0 [45,46] on the Genouest Galaxy server. First, reads were demultiplexed and merged, then chimera were removed using VSearch [47,48], and sequences were clustered using Swarm (aggregation parameter: d = 3) [46]. Singletons were filtered and removed from data. Finally, OTUs (operational taxonomic units) were taxonomically assigned using Blast [49] and the SILVA 138 (pintail 100) database. Contaminant OTUs present in the control samples (blanks, DNA extraction kit and lab reagents) were filtered and removed if their relative abundance reached more than 1% [34].

### 2.4. Statistical Analysis on Microbial Communities

Data (count table, OTUs table and samples data) were imported in R [50] for the analysis of the bacterial community composition and the diversity using the R packages 2 e *phyloseq* [51], *vegan* [52] and *ggplot2* [53]. Alpha diversity indices were calculated (Chao1 and Shannon’s diversity indices), and the means were compared according to the variables “species” and “sampling date” with the non-parametric test of Kruskal–Wallis (*p*-value threshold 0.05) and then pairwise comparisons with the Wilcoxon test (*p*-value adjust method holm). The beta-diversity analysis was built with the function decostand and the count transformation Hellinger [54,55,56], the Bray–Curtis distances were calculated with the function vegdist of the package *vegan* and the nMDS plot was drawn with the function metaMDS [57]. A permutational multivariate analysis of variance (PERMANOVA) was completed with the function Adonis [58] on these distances. Compositions of cœlomic microbiota were then compared at the phylum and order levels using R and Excel. A Venn diagram of shared and unique OTUs was built with the count table in Excel. Finally, the common core microbiota between the 3 species was determined using a prevalence threshold of 0.5 with the R package *microbiome* [59].

### 2.5. Enumeration and Isolation of Culturable Bacteria

First, 100 µL of pure and diluted (10^−1^) samples were deposited on marine agar media (Marine Broth 2216, Difco and European Bacteriological Agar, Biokar). Plates were incubated at 18 °C for 3 days before enumeration. Pairwise Wilcoxon tests were performed on culturable concentrations (threshold: 0.05). After bacterial enumeration, 831 strains of cœlomic bacteria were isolated in marine broth media (*H. forskali n* = 442, *M. glacialis* n = 343 and *S. granularis* n = 46). Finally, pure isolates were cryopreserved in glycerol 25% (*v*/*v*) in 96-well microplates and were stored at −80 °C before the antibacterial screenings.

### 2.6. Antimicrobial Activities of Cœlomic Isolates

Therefore, 6 pathogenic bacterial strains were selected as targets: *Lactococcus garvieae* (ATCC43921), *Listonella anguillarum* (NCBIM829), *Vibrio harveyi* (ORM4), *V. parahaemolyticus* (13028/A3), *V. tapetis* (CECT4600) and *Yersinia ruckeri* (ATCC29473). Strains were cultivated in marine broth media and incubated at 18 °C or 30 °C for 2 days. For the antibacterial assays, marine agar plates were inoculated by inundation with the pathogenic strains (10^6^ UFC/mL). Overnight cultures of cœlomic isolates in marine broth were deposited in 2 µL spot on the plate. A 2 µL spot of sterile marine broth was used as the negative control, and a 2 µL spot of kanamycin (1 mg/mL) as the positive control. Plates were incubated at 18 °C for 3 days, and potential antibacterial activity was visualized with Gel Doc XR (Bio-Rad, Hercules, CA, USA).

### 2.7. Identification of Antibacterial Isolates

Isolates exhibiting an antibacterial activity were selected. DNA was extracted with the NucleoSpin Microbial DNA kit from Macherey-Nagel (Düren, Germany), according to the manufacturer’s protocol. The 16S rDNA was amplified by PCR using the modified universal primers forward 24F-w18 (5′-GAGTTTGATCMTGGCTCAG-3′) and reverse 1492R-w20 (5′-GNTACCTTGTTACGACTT-3′) [60,61,62] and the PCR master mix from Promega (Promega, Madison, WI, USA), according to the following program: an initial step of denaturation at 95 °C for 5 min, then 30 cycles of 90 °C for 30 s, 54 °C for 1 min, 72 °C for 1 min and a final extension step of 5 min at 72 °C. PCR products were visualized by gel electrophoresis (1% agarose in 1X TAE buffer containing Sybr™ green, Promega, Madison, WI, USA). DNA concentration was quantified by fluorometries with the Qubit Fluorometer (Invitrogen, by ThermoFisher scientific, Waltham, MA, USA).

DNA products were sequenced by GATC (Eurofins genomics, Köln, Germany). Sequences were analyzed with BLASTn [63] on the NCBI server (https://blast.ncbi.nlm.nih.gov/Blast.cgi?PROGRAM=blastn&PAGE_TYPE=BlastSearch&LINK_LOC=blasthome, accessed on 31 January 2025) and aligned with similar sequences (taxonomical affiliation if % homology > 99).

## 3. Results

The cœlomic microbiota of 15 animals per species and per sampling were collected at the same time and the same place, resulting in 180 samples of cœlomic microbiota and 12 samples of seawater.

### 3.1. Sequencing Information and Quality Control

First, 3,088,247 paired-end sequences were assembled and preprocessed (mean length of 254 pb) and distributed as follows: 11,042,942 total amplicons for the sea cucumber *H. forskali*, 8,909,074 total amplicons for the sea star *M. glacialis*, 10,406,577 for the sea urchin *S. granularis* and 2,729,654 in seawater samples (Supplementary Table S1). The mean library depth reached 176,001 amplicons per sample, varied according to species: 184,049 amplicons for *H. forskali*, 159,091 for *M. glacialis*, 173,443 for *S. granularis* and 227,471 for seawater samples. After cleaning, filtering and clustering, the number of total sequences was 8,937,716 clustered in 17,101 OTUs. The cœlomic microbiota of *H. forskali*, *M. glacialis*, *S. granularis* and seawater samples were composed of 9927, 7696, 7424 and 2563 OTUs, respectively.

### 3.2. Composition of the Cœlomic Microbiota of the Three Echinoderms and of the Bacterial Communities of the Surrounding Sea Water

#### 3.2.1. *H. forskali*

**Figure 2:**
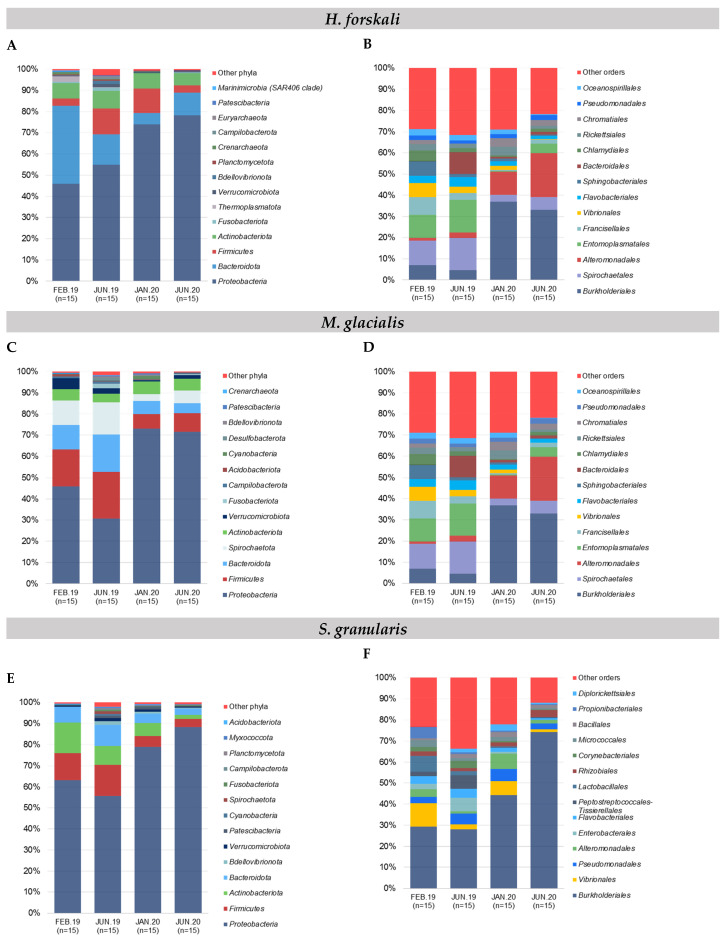

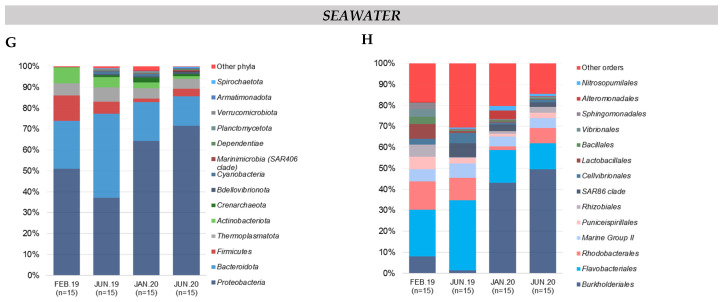
Relative composition of the major taxa present in the cœlomic microbiota of the 3 species and the bacterial communities of seawater according to sampling date. (**A**,**B**) Relative abundance in the 15 most abundant phyla and orders, respectively: (**A**,**B**) *H. forskali*; (**C**,**D**) *M. glacialis*; (**E**,**F**) *S. granularis*; (**G**,**H**) seawater samples. Sampling code: Feb.19 = February 2019, Jun.19 = June 2019, Jan.20 = January 2020 and Jun.20 = June 2020.

The composition of the cœlomic microbiota of *H. forskali* was overall dominated by the phylum Proteobacteria that represented more than a half of the total abundance, with a mean of 61.8% among the sampling dates (Figure 2A). The Bacteroidota, the Firmucutes and the Actinobacteroidota were secondary well represented. These four most abundant phyla accumulated more than 90% of the total abundance. Otherwise, the microbiota composition evolved over time, mainly between the samples from 2019 and 2020, with a general increase of the Proteobacteria, instead of the Bacteroidota. At a lower taxonomic rank, differences were accentuated between samplings (Figure 2B and Supplementary Table S2), showing very diverse and variable patterns of orders. Some orders represented high cumulative abundances, such as Burkholderiales (18.3%), Flavobacteriales (13.9%), Alteromonadales (9.9%), Vibrionales, Rhodobacterales and Pseudomonadales, and each reached more than 5% of the total abundance and exhibited temporal variations. The sampling in February 2019 was dominated by the Flavobacteriales and the Rhodobacterales and the samplings from 2020 by Burkholderiales. The increase in Burkholderiales and, to a lesser extent, Alteromonaldes in 2020 was concomitant and could explain the increase in the Gram-negative Proteobacteria at the phylum level, and in contrast, the Vibrionales and the Rhodobacterales decreased in 2020. For *H. forskali*, 23 orders with an abundance higher than 1% represented 82% of the total abundance, and 12 OTUs from seven main orders constituted 38% of the total abundance (Supplementary Table S2).

#### 3.2.2. *M. glacialis*

The Proteobacteria reached 54.1% of the total abundance in the cœlomic microbiota of *M. glacialis*, followed by Firmicutes (14.2%), Bacteroidota (10.1%) and Spirochaetota (9.5%) (Figure 2C). An important decrease in the Firmicutes, the Bacteroidota and the Spirochaetota was observed between the samplings from 2019 and 2020. In the rank order, the microbiota was mainly composed of the Burkholderiales (18.7%), the Spirochaetales (9.5%), the Alteromonadales (8.6%) and the Entomoplasmatales (8.4%) (Figure 2D). In 2019, the most abundant orders were the Spirochaetales and the Entomoplasmatales, shunted by the Proteobacteria orders of the Burkholderiales and the Alteromonadales in 2020 that dominated the microbiota with a mean cumulative abundance of 50.8%. More precisely, 13 OTUs from 10 orders accumulated 53.3% of the total abundance (Supplementary Table S2).

#### 3.2.3. *S. granularis*

The composition of the cœlomic microbiota of *S. granularis* was mainly composed of Proteobacteria (70.1%) and, to a lesser extent, of Firmicutes (9.6%), Actinobacteriota (9.6%) and Bacteroidota (6.6%) (Figure 2E). More precisely, at the rank order, the cœlomic microbiota of the sea urchin S. granularis was composed of Burkholderiales (from 28.7% in 2019 to 59.2% in 2020) and less abundant orders such as the Vibrionales (4.7%) or the Pseudomonadales (4.3%) (Figure 2F). Regarding the composition in OTUs, six OTUs (5 orders) represented 48.2% (Supplementary Table S2).

#### 3.2.4. Composition of Bacterial Communities of the Surrounding Seawater

In samples of seawater, the relative abundance of the main phyla evolved according to time, with a massive increase of the *Proteobacteria* coupled with a decrease of the *Bacteroidota,* the *Firmicutes* and the *Actinobacteria* and a stable abundance of the *Thermoplasmatota* (mean abundance of 5.2%) (Figure 2G). In samples of seawater, four orders constituted the majority of the total abundance (63.1%): the *Burkholderiales* (32.1%), the *Flavobacteriales* (18%), the *Rhodobacterales* (7.8%) and the Marine Group II (5.2%,) (Figure 2H). The order *Burkholderiales* (phylum *Proteobacteria*) and the order *Flavobacteriales* (phylum *Bacteroidota*) dominated the composition in the seawater samples and showed an important increase in 2020. Finally, 18 OTUs (seven orders) contributed 52.7% of the total abundance in the samples of seawater (Supplementary Table S2).

### 3.3. Diversities (Alpha and Beta Analyses)

#### 3.3.1. α Diversity

**Figure 3:**
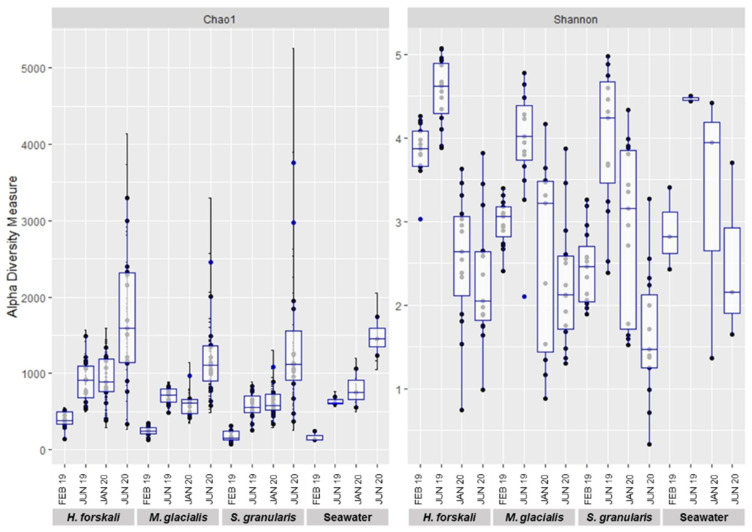
Plot of alpha-diversity comparison of the cœlomic microbiota of 3 species of Echinoderms: *H. forskali*, *M. glacialis*, *S. granularis* and seawater.

Alpha-diversity was analyzed to compare the intrinsic diversity in each type of sample. Analysis of intraspecific richness (Chao1 richness estimator) and diversity (Shannon’s diversity index) exhibited differences between species and date of sampling (Figure 3) and were confirmed by the results of the Kruskal–Wallis test (*p*-value threshold 0.05) (Table 1 and Supplementary Table S3). The diversity of the cœlomic microbiota highly varied according to time, whatever the species concerned. The highest values of the Shannon’s diversity index were observed in June 2019 and the lowest in June 2020. The estimated richness evolved according to time to a lesser extent, and in contrast, the richness increased in June 2020. The comparison of the three species of Echinoderms demonstrated that the microbiota of *H. forskali* exhibited the highest number of OTUs, estimated richness and diversity for each date of sampling (Figure 3 and rarefaction curve Supplementary Figure S2).

#### 3.3.2. Diversity Analysis

**Figure 4:**
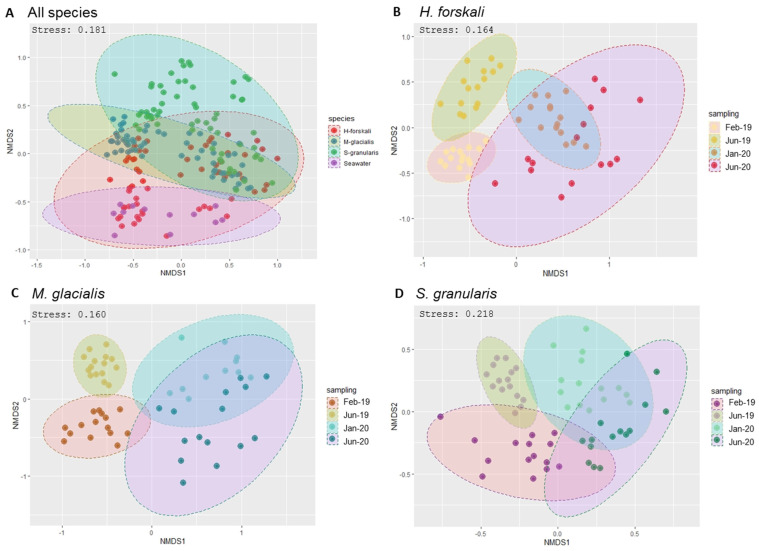
Bacterial community dissimilarities between the 3 species of Echinoderms and the sampling dates. Non-metric multidimensional scaling (nMDS) plot based on the Bray–Curtis distances with a 0.95 interval confidence ellipse. (**A**) All Echinoderms samples; (**B**–**D**) within samples of cœlomic fluid microbiota of *H. forskali* (**B**), *M. glacialis* (**C**) and *S. granularis* (**D**). Sampling code: Feb_19 = February 2019, Jun_19 = June 2019, Jan_20 = January 2020 and Jun_20 = June 2020.

Beta-diversity analysis was assessed using the Bray–Curtis distance metrics (Figure 3). First, samples were grouped by species (Figure 4A) and also by the date of sampling, with the nMDS plots built per species (Figure 4B–D), showing temporally variable microbiota in the three species of Echinoderms. The results of the PERMANOVA analysis validated significant differences between the composition and abundance in the bacterial communities according to these variables: species and date sampling (Table 2). Thus, the microbial communities in the cœlomic microbiota of the three Echinoderms varied according to time and differed from one species to another one.

#### 3.3.3. OTUs Distribution and Core Microbiota Among the Three Echinoderms

**Figure 5:**
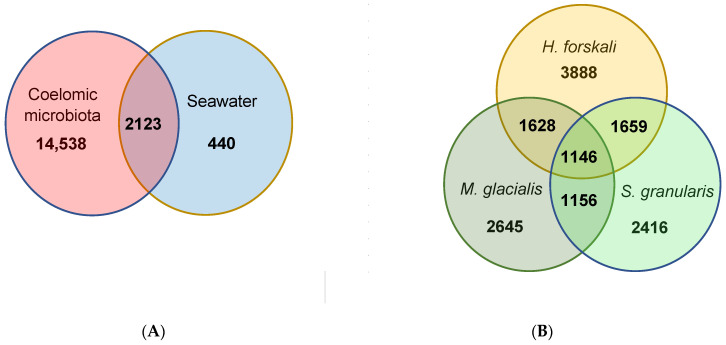
Venn diagrams of shared and unique OTUs. (**A**) Shared and unique OTUs between the total cœlomic microbiota and the seawater; (**B**) shared and unique OTUs between the cœlomic microbiota of the 3 Echinoderms.

The total cœlomic microbiota of the three echinoderms was composed by 16,661 OTUs; among which, only 2123 shared OTUs (12.7%) with the bacterial communities of seawater (Figure 5A). In total, 87.3% of the total OTUs were unshared from the cœlomic fluid microbiota of the three Echinoderms and represented a total abundance of 24.2%. These OTUs mainly belonged to families such as *Francisellaceae* or *Flavobacteriaceae* (Table 3).

From these cœlomic OTUs, the three species of Echinoderms shared 1146 OTUs, which represented 11.5% of the total OTUs in *H. forskali*, 14.9% in *M. glacialis* and 15.4% in *S. granularis* (Figure 5B). Otherwise, *H. forskali* contained the highest proportion of unique OTUs (39.2% of OTUs abundance and 8.6% of the sequences), followed by *M. glacialis* (34.4% of the OTU abundance and 7.2% of the sequences), *S. granularis* (32.5% of the OTU abundance and 8.7% of the sequences) and the seawater (17.2%) (Figure 5 and Table 3).

The main abundant families in the cœlomic fluid of *H. forskali* were the *Flavobacteriaceae* with 260 OTUs and, especially, the genera *Flavobacterium, Formosa* and *Lutibacter;* the *Chitinophagaceae* and the *Arenicellaceae* (Table 3). For *M. glacialis*, the OTUs belonged to families such as *Francisellaceae* (49 OTUs), *Diplorickettsiaceae* (57 OTUs), *Flavobacteriaceae* (59 OTUs) and *Spirochaetaceae* (63 OTUs) and, for *S. granularis*, *Diplorickettsiaceae* (78 OTUs), *Legionellaceae* (76 OTUs) and also *Flavobacteriaceae* (49 OTUs).

**Figure 6:**
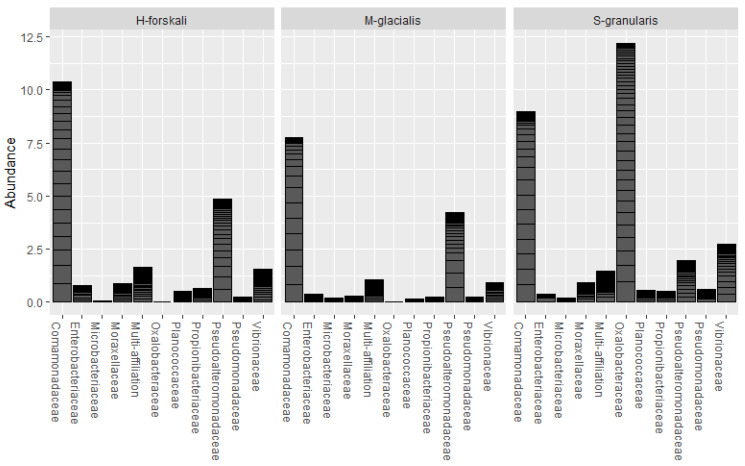
Relative abundance of the core microbiota of the 3 Echinoderms from the Glénan Archipelago.

The common core microbiota of the three species of Echinoderms was determined (prevalence threshold 0.5). This core was constituted by 10 families and 16 OTUs (Figure 6); among which, the *Comamonadaceae*, the *Pseudoalteromonadaceae*, the *Oxalobacteraceae* and the *Vibrionaceae* represented widespread marine families. The relative abundance of these core taxa differed between the three species.

### 3.4. Culturable Microbiota

#### 3.4.1. Enumeration of Culturable Microbiota

**Figure 7:**
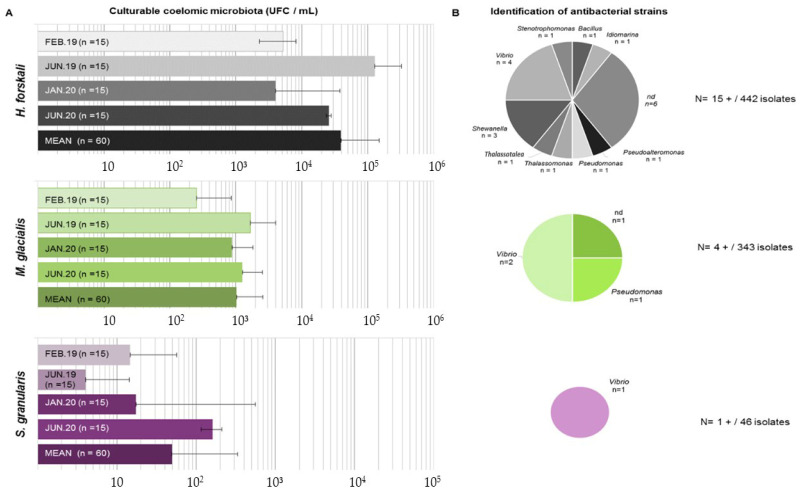
Results of the culturable microbiota assays. (**A**) Enumeration of culturable microbiota (in CFU/mL) for the 3 species according to sampling (standard deviation among the samples indicated by horizontal bars); (**B**) identification of antibacterial strains isolated from culturable microbiota for each species.

After incubation, the colonies were counted on marine agar plates to determine the concentration of culturable cœlomic microbiota (Figure 7A). The highest concentration of culturable microbiota was found for *H. forskali* with a mean of 3.9 × 10^4^ CFU/mL, while the culturable microbiota of *M. glacialis* and *S. granularis* were estimated at 1.0 × 10^3^ CFU/mL and 4.9 × 10^1^ CFU/mL, respectively. The culturable concentration of bacteria in seawater was 8.1 × 10^2^ CFU/mL. The results of the statistical tests exhibited significant differences in term of concentrations of culturable microbiota between species (Kruskal–Wallis test with a threshold of 0.05, chi-squared of 142.25, *p*-value < 2.2 × 10^−16^) and a seasonal difference in the cœlomic culturable microbiota in *H. forskali* (pairwise comparison using the Wilcoxon test, threshold 0.05, *p*-value 1.50 × 10^−6^) and in *M. glacialis* (pairwise comparison using the Wilcoxon test, threshold 0.05, *p*-value 0.00295).

#### 3.4.2. Screening for Antibacterial Activity in Culturable Strains

Therefore, 831 isolates were assayed for antimicrobial activity, from which 442, 343 and 46 originated from the cœlomic fluids of *H. forskali*, *M. glacialis* and *S. granularis,* respectively (Figure 7B). Among these bacterial strains, only 20 presented a positive antibacterial activity against one or more pathogens, representing 2.4% of the total isolates (Figure 6). After cryopreservation at −80 °C, 6 of the 20 antibacterial strains did not spread. *H. forskali*, which showed the highest concentration in culturable bacteria, also contained the highest number of antibacterial strains [15]. The 16S identification of the strains showed different genera (Figure 6), mainly *Vibrio* and *Shewanella*.

## 4. Discussion

Cœlomic microbiota of three species from three different classes of Echinoderms collected in the same habitat were sampled for 2 years: the detritivorous sea cucumber *H. forskali*, the carnivorous sea star *M. glacialis* and the herbivorous sea urchin *S. granularis*. The cœlomic microbiota of these three species were analyzed both by a culture-independent (metabarcoding 16S) and by a culture-based approach to study the composition and their antibacterial activities of the cœlomic microbiota.

### 4.1. Richness and Diversity of the Microbiota Among the Three Echinoderms and the Bacterial Communities of the Surrounding Seawater

The results of the microbiota diversity exhibited a variable diversity and also richness according to species and sampling date, with the highest alpha diversity for *H. forskali*, whatever the date of sampling, compared to the other Echinoderms and the bacterial communities of the seawater. These results were completed with sequencing information such as library depth and clustering process with the number of OTUs formed for *H. forskali* and also with the enumeration of culturable bacteria, where the concentration for *H. forskali* (3.9 × 10^4^ CFU/mL) was 10- and 1000-fold higher than that for *M. glacialis* and *S. granularis*, respectively. The variations in diversity and richness across Echinoderm species have already been recorded in other areas [10,38,40]. Moreover, the alpha-diversity rates varied between the samplings, suggesting a temporal variation of the cœlomic microbiota in term of richness and diversity.

The cœlomic microbiota was dominated by the Gram-negative phylum Proteobacteria, which represented more than half of the total abundance, and also by the Bacteroidota, the Firmicutes and the Actinobacteriota. These three phyla reached more than 80% of the cumulative abundance whatever the species. Differences between species at the phylum rank could be observed between the abundance of secondary phyla or less abundant phyla, such as Spirochaetota and Verrucomicrobiota for *M. glacialis*, and the Thermoplasmatota for the seawater samples. The Proteobacteria constituted the main phylum in a marine environment, where they played a key role, especially in the recycling of dissolved organic matter, associated with Bacteroidota [25,64,65,66,67]. These more abundant phyla were also in the majority in the microbiota of marine invertebrates and occupied the same abundance ranks in the cœlomic microbiota of other Echinoderms [38,40,68] or in other marine invertebrates such as Ascidians [69].

These variations were also observed at a lower taxonomic rank: the pattern of relative abundance of the main orders differed according to species. For each species of echinoderms, only four orders cumulated more than 50% of the abundance, varying according to species between Burkholderiales, Flavobacteriales, Alteromonadales, Vibrionales, Entomoplasmatales and Rhodobacterales. These orders were frequently found in marine environments and marine host microbiota. They occupied different functions, from organic compounds degradation [70] to the production of antibacterial peptides [71,72] or, conversely, were responsible for pathogenic reactions [73]. Within these orders, only a few OTUs contributed to explaining the main abundance. Thus, the cœlomic microbiota of the three Echinoderms were constituted by only a few OTUs that represented the main abundance and occupied the niches; nevertheless, the diversity between Echinoderms species was explained by numerous sub-abundant OTUs. Differential compositions in the abundant and sub-abundant taxa and their roles have already been described in microbiome studies [74,75,76,77]. The abundance of a few OTUs was observed in other Echinoderms, such as the sea star *Asterias amurensis,* where one genus related to *Helicobacter* reached 97.3% of the reads [40], and also the abundance of the family *Flavobacteriaceae* in Holothurians [10,22,78].

The beta-diversity analysis built with the Bray–Curtis distances showed that the microbiota of the three Echinoderms were distinct between them but also between sampling dates. This suggested that the microbiota varied across time and host and that the environment played a role in microbiota selection [79,80]. Otherwise, temporal variations of the cœlomic microbiota of the three echinoderms and the bacterial communities of the seawater were assessed, with a global increase of Proteobacteria in 2020 probably correlated with the increase of the Burkholderiales, validating the temporal variations previously deduced by richness and diversity analyses. The temporal variations in the composition of the microbiota were highly documented for gut microbiota in vertebrates and correlated with health or food habits [81] but remain largely unexplored for marine invertebrates. Even if the cœlomic compartment seemed further away from the environment and more stable in the face of environmental fluctuations [23], the cœlomic fluid and its microbiota were subject to temporal variations that could be explained by different factors such as host physiology, health, feeding behavior or other environmental factors coming from their natural habitat [79,82].

Many unshared OTUs were found in the cœlomic microbiota of the three Echinoderms and in the bacterial communities of seawater. Some scientists used the term “specific” to qualify such OTUs and therefore the bacterial community found in one organism [83]. Is this term well adapted or appropriated? The highest number and proportion of unshared and “specific” OTUs were found in the cœlomic microbiota of *H. forskali,* notably with the abundant families *Flavobacteriaceae (Bacteroidota)* represented by 260 OTUs and genera such as *Flavobacterium* (39 OTUs) or *Tenacibaculum* (16 OTUs). These genera were often associated with fish disease [73,84,85,86], even if other genera were correlated with the organic degradation of algal polysaccharides such as the genera *Formosa* (four OTUs) [87]. In the cœlomic microbiota of the sea star *M. glacialis*, the OTUs were significantly different and belonged mainly to the families *Francisellaceae, Hyphomonadaceae* or *Spirochaetaceae* and in the sea urchin *S. granularis* from *Diplorickettsiaceae* and *Legionellaceae*. Unshared OTUs could take part in a “specific microbiota” for each species of the three Echinoderms because of their presence and abundance correlated with one specific host [34,88]. The shared OTUs between the three species could also be qualified as a “common specific microbiota”.

### 4.2. Core Microbiote

Otherwise, defining a core microbiota was a complex choice and weighing between parameters such as prevalence, abundance, function and specificity related to a host, and the methodology was still relevant [89,90,91,92,93,94]. Moreover, the core microbiota could be categorized on a multiscale: from one specific host to a specific population in an ecological niche [92] or species living in a same habitat, which should be omnipresent [79] or variable by time [92]. The “common core microbiota” of the three Echinoderms, constituted by the most prevalent taxa, was composed by ubiquitous taxa for this group of Echinoderms living in a same habitat and, indeed, probably associated with an ecological niche [95].

These observations confirmed the conclusion that the main part of the cœlomic microbiota from these three Echinoderms species were highly variable and responsible for diversity and that cœlomic microbiota was constituted by a few abundant OTUs dominating the total abundance. Another hypothesis suggested by the presence of “specific” OTUs only found in the cœlomic microbiota of Echinoderms and absent from seawater was the selection of the microbiota. Who and how was this selection made: by the host, by the microbiota itself or by the holobiont [96]? A new species in the *Pseudoalteromonas* genus was recently described, and bacterial strains were isolated from the coelomic fluid of the sea cucumber *H. forskali* and from the surrounding seawater of the animal sampling site, respectively [97].

### 4.3. Antibacterial Activities from the Bacterial Microbiota

From the culturable microbiota that represented a weak part of the total microbiota [23], bacterial strains were isolated and cultivated, 2.4% of which exhibited an antibacterial activity against pathogenic bacterial strains in aquaculture. These strains belonged to abundant taxa present in the metabarcoding data: first, the genus *Pseudoalteromonas* with a total abundance of 6.3% and a total of 512 OTUs in the cœlomic microbiota of the three echinoderms and then *Vibrio* (1.6%, 144 OTUs), *Shewanella* (0.1%, 26 OTUs), *Idiomarina* (0.02%, 6 OTUs), *Thalassotalea* (0.1%, 22 OTUs), *Pseudomonas* (1.2%, 108 OTUs) and *Bacillus* (0.3%, 57 OTUs). These genera were common culturable bacteria present in the cœlomic fluid of Echinoderms [10,22] and in the marine environment. Among these genera, some are associated with antibacterial activities and often known to produce antibacterial peptides, such as the genus *Pseudoalteromonas* [11,71,72], with a growing performance as probiotics such as *Bacillus* sp. [14,21] or, in contrast, associated with disease in the aquaculture, such as the genera *Vibrio* and *Shewanella* [98,99]. The isolation of antibacterial strains from Echinoderms was already related to sea urchins [40] or to sea cucumbers [10,11]. The presence of these abundant antibacterial or interesting strains supports the role of the microbiota in the host health [82] and also the role of the cœlomic compartment in Echinoderm immunity [4,5,100].

## 5. Conclusions

The cœlomic microbiota of three species of Echinoderms living in a same habitat: the sea cucumber *H. forskali*, the sea star *M. glacialis* and the sea urchin *S. granularis* were analyzed for two years in the Glénan Archipelago (South Brittany, France). The results obtained with metabarcoding 16S revealed a composition of shared and unshared bacterial communities within the cœlomic microbiota of the three Echinoderms on the one hand and, on the other hand, a very highly variable composition over time, thus showing a plasticity of cœlomic microbiota of each studied echinoderm. The bacterial community in the cœlomic fluid of the three echinoderms appears mainly to be distinct from seawater. The composition was dominated by Proteobacteria overall and by only a few OTUs, which constituted the majority of the total abundance. The presence of numerous “specific” OTUs and of antibacterial strains in the cœlomic microbiota of the three Echinoderms suggested a selection of these microbiota. Furthermore, the culturable strains exhibiting antibacterial activities isolated from Echinoderms offered new perspectives for biotechnological issues as potential probiotic candidates.

## Figures and Tables

**Table 1:** Statistical analysis on the alpha-diversity indices (Kruskal–Wallis test, threshold 0.05).

	Chao1’s Richness		Shannon’s Diversity	
Variables	Chi-Squared	*p*-Value	Chi-Squared	*p*-Value
species	12.072	0.00714 *	8.8245	0.03172 *
date of sampling	122.4	<2.2 × 10^−16^ *	90.096	<2.2 × 10^−16^ *
species + date of sampling	137.37	<2.2 × 10^−16^ *	112.04	<2.2 × 10^−16^ *

* Significant for *p*-value < threshold alpha 0.05.

**Table 2:** Permutational multivariate analysis of THE variance (PERMANOVA) using the Bray–Curtis distance.

Variables	F	R^2^	*p*-Value	
Species	4.1	0.05454	0.001	*
Date of sampling/				
*H. forskali*	6.8796	0.2693	0.001	*
*M. glacialis*	5.9295	0.25489	0.001	*
*S. granularis*	5.002	0.21133	0.001	*

* Significant for *p*-value < threshold alpha 0.05.

**Table 3:** Table of the 15 most abundant families of the unshared OTUs by species. “Total unshared” represented the mean of the unshared count abundance and of the unshared OTUs.

*H. forskali*			*M. glacialis*			*S. granularis*			Echinoderms		
Families	Abundance (%)	Nb. OTUs	Families	Abundance (%)	Nb. OTUs	Families	Abundance (%)	Nb. OTUs	Families	Abundance (%)	Nb. OTUs
*Flavobacteriaceae*	0.34%	260	*Francisellaceae*	0.29%	49	*Diplorickettsiaceae*	0.55%	78	*Francisellaceae*	1.19%	69
*Chitinophagaceae*	0.31%	36	*Diplorickettsiaceae*	0.27%	57	*Legionellaceae*	0.43%	76	*Flavobacteriaceae*	0.96%	464
*Arenicellaceae*	0.22%	13	*Hyphomonadaceae*	0.26%	14	*Chitinophagaceae*	0.41%	27	*Simkaniaceae*	0.78%	88
*Sphingobacteriaceae*	0.20%	37	*Prevotellaceae*	0.24%	11	*Spirochaetaceae*	0.38%	3	*Fusobacteriaceae*	0.54%	36
*Legionellaceae*	0.17%	46	*Legionellaceae*	0.22%	53	*Hymenobacteraceae*	0.25%	19	*Legionellaceae*	0.53%	221
*Hymenobacteraceae*	0.17%	20	*Bacillaceae*	0.21%	22	*Bdellovibrionaceae*	0.17%	21	*Diplorickettsiaceae*	0.51%	218
*Sphingomonadaceae*	0.17%	48	*Spirochaetaceae*	0.21%	63	*Prevotellaceae*	0.14%	14	*Spirosomaceae*	0.44%	61
*Orbaceae*	0.16%	1	*Flavobacteriaceae*	0.20%	59	*Flavobacteriaceae*	0.14%	49	*Spirochaetaceae*	0.44%	90
*Diplorickettsiaceae*	0.16%	35	*Fokiniaceae*	0.18%	3	*Parachlamydiaceae*	0.13%	39	*Sphingomonadaceae*	0.43%	211
*Rhodobacteraceae*	0.15%	137	*Endozoicomonadaceae*	0.16%	29	*Gemmatimonadaceae*	0.12%	9	*Holosporaceae*	0.42%	36
*Bdellovibrionaceae*	0.15%	32	*Nitrososphaeraceae*	0.15%	2	*Sphingomonadaceae*	0.10%	35	*Nocardioidaceae*	0.42%	85
*Rhizobiaceae*	0.13%	477	*Ruminococcaceae*	0.15%	10	*Bryobacteraceae*	0.10%	4	*Rhodobacteraceae*	0.40%	249
*Cyclobacteriaceae*	0.12%	23	*Prolixibacteraceae*	0.14%	41	*Acetobacteraceae*	0.10%	9	*Chitinophagaceae*	0.39%	103
*Micropepsaceae*	0.12%	3	*Marinifilaceae*	0.14%	34	*Spirosomaceae*	0.10%	7	*Sphingobacteriaceae*	0.39%	76
*Nitrincolaceae*	0.12%	11	*Chitinophagaceae*	0.12%	21	*Cellvibrionaceae*	0.09%	5	*Rickettsiaceae*	0.36%	29
Total unshared	8.6%	39.2%	Total unshared	7.2%	34.4%	Total unshared	8.7%	32.5%	Total unshared	24.2%	87.3%

## Data Availability

The original contributions presented in the study are included in the article, further inquiries can be directed to the corresponding authors.

## Supplementary Materials

The following supporting information can be downloaded at https://www.mdpi.com/article/10.3390/biology14040430/s1: Supplementary Figure S1: Mean weight of the 3 species of Echinoderms according to the sampling period. Supplementary Figure S2: Rarefaction curves. Supplementary Table S1: Results of the sequencing data preprocess. Supplementary Table S2: Relative abundance and OTUs content of the 15 first orders for the 3 Echinoderms. Supplementary Table S3: Kruskal–Wallis test on the alpha-diversity measures for the 3 species of Echinoderms. Supplementary Table S4: Statistical analysis on bacterial enumeration (Kruskal–Wallis test, *p*-value threshold 0.05).
