# Supplementary material for: The Cœlomic Microbiota Among Three Echinoderms: The Black Sea Cucumber Holothuria forskali, the Sea Star Marthasterias glacialis, and the Sea Urchin Sphaerechinus granularis
Source: Biology (Basel). 2025 Apr 16;14(4):430. doi: 10.3390/biology14040430 (PMC12024532; doi:10.3390/biology14040430)
Supplement: Supplementary file 1 [file biology-14-00430-s001.zip › biology-3553121-figure.pdf]

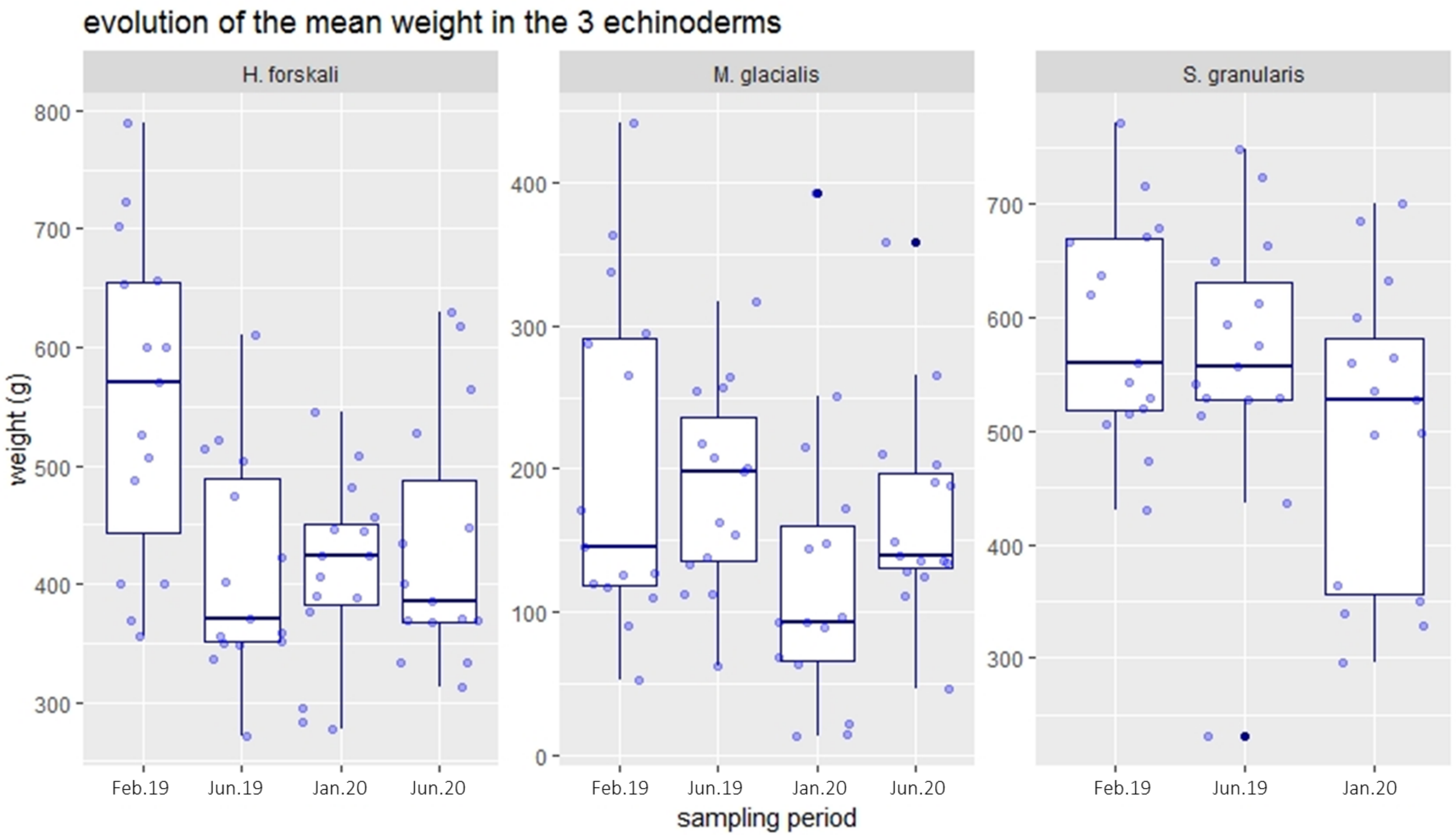

Supplementary figure 1: mean weight of the 3 species of Echinoderms according to the sampling period.

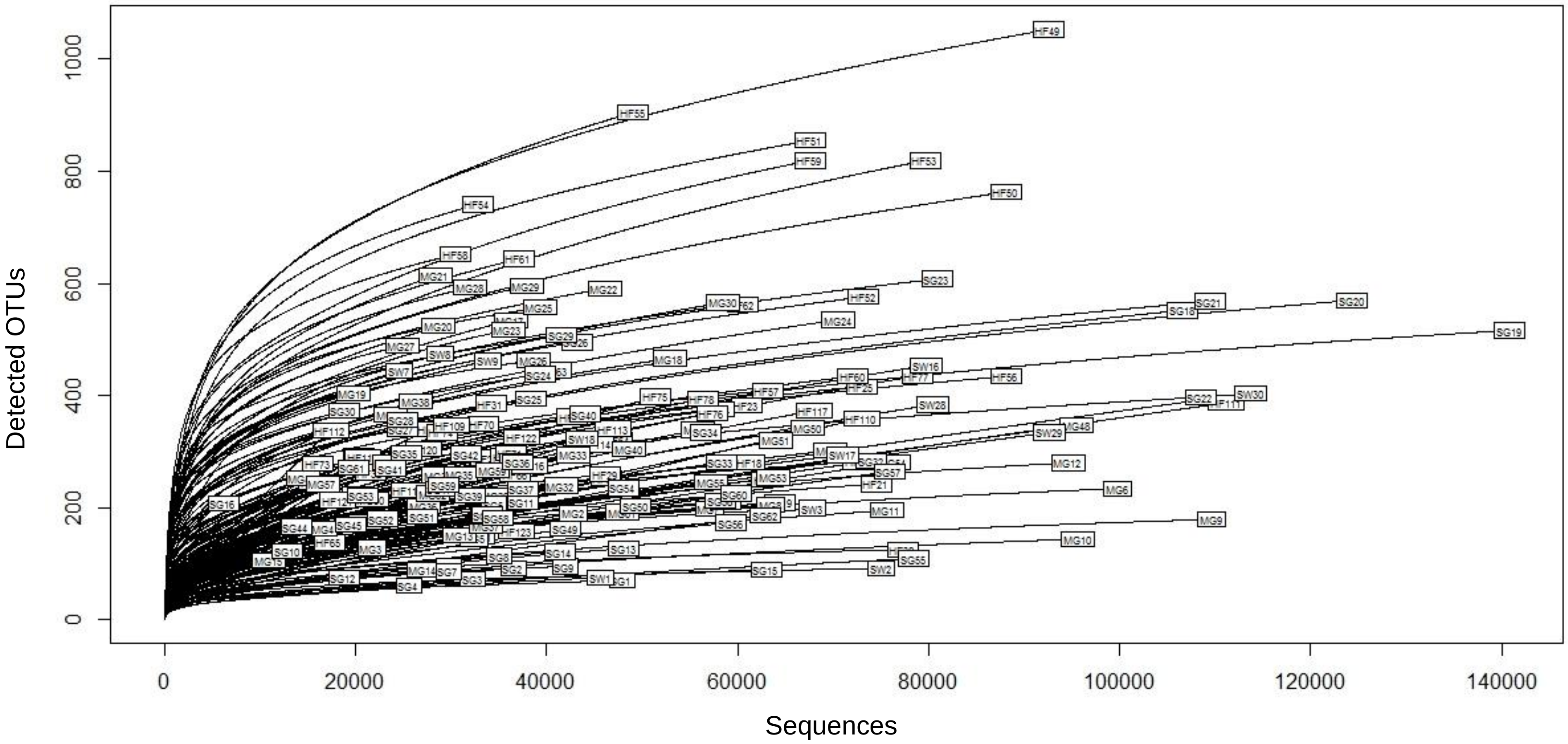

Supplementary figure 2: rarefaction curves.
