# Supplementary material for: The Cœlomic Microbiota Among Three Echinoderms: The Black Sea Cucumber Holothuria forskali, the Sea Star Marthasterias glacialis, and the Sea Urchin Sphaerechinus granularis
Source: Biology (Basel). 2025 Apr 16;14(4):430. doi: 10.3390/biology14040430 (PMC12024532; doi:10.3390/biology14040430)
Supplement: Supplementary file 1 [file biology-14-00430-s001.zip › biology-3553121-table.pdf]

Supplementary table 1 : Results of sequencing data preprocess

Process	Demultiplexing-merging	Clustering	removing Chimera	Tax. affiliation	Data for analysis (after removing singletons and contaminants)	
Species / type	paired-end assembled sequences	Sequences	Sequences	Sequences	Sequences	OTUs
Holothuria forskali	11 042 942	6 437 256	6 411 318	6 302 164	2 948 191	9 927
Marthasterias glacialis	8 909 074	4 327 330	4 259 867	4 200 360	2 493 538	7 696
Sphaerechinus granularis	10 406 577	3 516 116	3 389 431	3 330 994	2 738 863	7 424
seawater	2 729 654	309 801	301 858	289 832	757 124	2 563
**Total**	**33 088 247**	**14 590 503**	**14 362 474**	**14 123 350**	**8 937 716**	**17 101**

Supplementary table 2: relative abundance and OTUs content of the 15 first orders for the 3 echinoderms

H. forskali			
Orders	Relative abundance (%)	number of OTUs	Number of OTUs with abundance >1%
Alteromonadales	9.9%	633	**1**
Bacillales	1.9%	94	0
Burkholderiales	18.3 %	731	**2**
Caulobacterales	1.3%	71	0
Corynebacteriales	1.8%	137	0
Cytophagales	1.3%	106	0
Enterobacterales	3.2%	96	**2**
Flavobacteriales	13.9 %	706	**2**
Lactobacillales	1.2%	114	0
Peptostreptococcales T.	1.9%	84	0
Propionibacteriales	1.3%	94	0
Pseudomonadales	4.4%	277	**1**
Rhizobiales	1.8%	2713	0
Rhodobacterales	5.0%	287	**2**
Vibrionales	5.6%	221	**2**
**Total**	**72.8 %**	**6364**	**38.0%**

M. glacialis			
Orders	Relative abundance (%)	number of OTUs	Number of OTUs with abundance >1%
Alteromonadales	8.6%	373	**1**
Bacteroidales	2.9%	215	0
Burkholderiales	18.7 %	577	**2**
Caulobacterales	1.6%	62	0
Chlamydiales	2.7%	125	**1**
Chromatiales	2.1%	22	**1**
Entomoplasmatales	8.4%	80	**1**
Flavobacteriales	3.0%	274	0
Francisellales	4.1%	65	**1**
Oceanospirillales	2.0%	68	**1**
Pseudomonadales	2.1%	149	0
Rickettsiales	2.4%	103	0
Sphingobacteriales	2.9%	85	**1**
Spirochaetales	9.5%	74	**2**
Vibrionales	3.2%	161	**2**
**Total**	**74.1 %**	**2433**	**53.3%**

S. granularis			
Orders	Relative abundance (%)	number of OTUs	Number of OTUs with abundance >1%
Alteromonadales	3.1%	220	**1**
Bacillales	1.7%	78	0
Bacteroidales	1.4%	125	0
Burkholderiales	43.1%	634	**2**
Corynebacteriales	1.8%	118	0
Diplorickettsiales	1.4%	122	0
Enterobacterales	2.9%	76	**1**
Flavobacteriales	2.7%	221	0
Lactobacillales	2.4%	128	0
Micrococcales	1.7%	222	0
Peptostreptococcal es-T.	2.6%	84	0
Propionibacteriales	1.6%	85	0
Pseudomonadales	4.3%	235	**1**
Rhizobiales	2.1%	2184	0
Vibrionales	4.7%	109	**1**
**Total**	**77.6%**	**4641**	**48.2%**

**Seawater**			
Orders	Relative abundance (%)	number of OTUs	Number of OTUs with abundance >1%
Alteromonadales	1.1%	57	0
Bacillales	1.3%	21	0
Burkholderiales	32.1%	252	**2**
Cellvibrionales	2.0%	54	0
Flavobacteriales	18.0%	322	**4**
Lactobacillales	2.0%	28	**1**
Marine Group II	5.2%	13	**2**
Nitrosopumilales	1.1%	5	0
Pseudomonadale s	0.9%	51	0
Puniceispirillales	3.2%	35	1
Rhizobiales	2.7%	732	0
Rhodobacterales	7.8%	107	**4**
SAR86 clade	2.4%	22	**4**
Sphingomonadale s	1.1%	34	0
Vibrionales	1.2%	16	0
**Total**	**82.1%**	**1749**	**52.7%**

Supplementay table 3: Kruskal-Wallis test on the alpha Alpha diversity measures, for the 3 species of echinoderms.

Indices and variable	Chao1 ~ Sampling			Shannon ~ Sampling		
**Species**	**chi-squared**	**p-value**		**chi-squared**	**p-value**	
H. forskali	34.185	1.81E-07	*	46.12	5.35E-10	*
M. glacialis	42.449	3.22E-09	*	25.684	1.11E-05	*
S. granularis	41.646	4.77E-09	*	30.117	1.30E-06	*
Seawater	9.4615	0.02374	*	6.6923	0.08238	

* significant for p-value < threshold alpha 0.05

Supplementary table 4: Statistical analysis on bacterial enumeration (Kruskal-Wallis test, p-value threshold 0.05)

Variables	chi-squared	p-value
species	142.25	< 2.2e-16*
species + season	151.92	< 2.2e-16*

* significant for p-value < threshold alpha 0.05
